# How Mandatory Can We Make Vaccination?

**DOI:** 10.1093/phe/phac026

**Published:** 2022-12-21

**Authors:** Ben Saunders

**Affiliations:** University of Southampton, Southampton, SO17 1BJ, UK

## Abstract

The novel coronavirus (SARS-CoV-2) pandemic has refocused attention on the issue of mandatory vaccination. Some have suggested that vaccines ought to be mandatory, while others propose more moderate alternatives, such as incentives. This piece surveys a range of possible interventions, ranging from mandates through to education. All may have their place, depending on circumstances. However, it is worth clarifying the options available to policymakers, since there is sometimes confusion over whether a particular policy constitutes a mandate or not. Further, I illustrate a different kind of alternative to mandatory vaccination. Rather than seeking less coercive alternatives to a mandate, we might instead employ an alternative mandate, which requires people to do something less than get vaccinated. For instance, we might merely require people to attend an appointment at a vaccine clinic. Whether this mandatory attendance policy is justified will depend on specific circumstances, but it represents another way to promote vaccination, without mandating it. In some cases, this may represent an appropriate balance between promoting public health goals and respecting individual liberty.

## Introduction

In August 2020, Australian Prime Minister Scott Morrison told Melbourne radio station 3AW that he expected the Covid-19 vaccination to be ‘as mandatory as you could possibly make it’ allowing only for medical exemptions (Worthington, 2020). But, later the same morning, he told Sydney radio station 2GB that it was ‘not going to be compulsory to have the vaccine’ (Hayne and Norman, 2020). This was widely seen as backtracking on his earlier comments, though some authors do distinguish mandates from compulsion, such that vaccination could be mandatory but not compulsory.

In fact, there are at least two distinctions between mandates and compulsion found in the literature. First, Navin and Largent (2017: 255–6) use ‘mandatory’ for policies imposing lack of access to goods and services, such as exclusion from state schools or benefits, reserving ‘compulsory’ for policies that involve criminalisation. This distinction is sometimes followed by others (e.g. Giubilini and Savulescu, 2019: 238; Williams, 2022: 2), though not universally (e.g. Savulescu *et al.*, 2021: 11). Second, Offit (2015 [2011]: 139) attributes a different distinction to Walter Orenstein, according to which ‘mandatory vaccination’ again implies denial of social privileges for refusers, but ‘compulsory vaccination’ means that ‘people who refuse are forcibly vaccinated’. Since forcible vaccination differs from criminal punishment, this distinction is not equivalent to that coined by Navin and Largent. A May 2022 policy brief from the World Health Organization follows this latter usage, noting that ‘Despite its name, “mandatory vaccination” is rarely compulsory, i.e., people are not forced to be vaccinated’ (WHO, 2022: 1).

If Morrison were invoking some such distinction, then his comments may have been consistent. Vaccination could be mandatory, but not compulsory (in one or other of these senses). However, his comments were, at least, confusing. Though sometimes distinguished, the terms ‘mandatory’ and ‘compulsory’ are often used interchangeably (Savulescu *et al.*, 2021: 11; Williams, 2022: 2). This is true both in ordinary, everyday speech (as implicitly admitted by WHO’s ‘Despite its name…’) and in academic discussions of vaccination (e.g. Flanigan, 2014; Camilleri, 2019: 249, n. 35; Kennedy, 2020) and other cases of government mandate/compulsion (e.g. Power, 2009: 99, n. 3; Lundell, 2012: 221; Chapman, 2019: 101, n. 1). For the avoidance of doubt, here I use ‘mandatory vaccination’ and ‘compulsory vaccination’ as equivalent terms. Further, I use both to refer to an authoritative requirement (legal or otherwise). If vaccination is mandated, then those who refuse the vaccine act wrongly.^1^

To be sure, policies are sometimes referred to as ‘mandatory’, even though they are not strictly so. This broader usage may be for rhetorical effect (Wynia, 2007). However, this wider use creates confusion between genuine mandates (which impose requirements or obligations) and other policies that are better characterised as (dis)incentive schemes, because they merely impose a cost on the non-vaccinated but do not require vaccination. There are various alternatives to mandates, including education, persuasion, nudges and incentives, which seek to promote the same end (vaccination) but use less coercive methods to do so. Thus, debates over mandatory vaccination often focus on whether mandates are necessary in order to achieve sufficient levels of vaccination, or whether this target can instead be achieved by other means, such as incentive payments (e.g. Savulescu, 2021). However, there is another kind of alternative.

Rather than employing interventions that stop short of a mandate, such as nudges or incentives, we could instead impose a different mandate, requiring people to do something less than getting vaccinated. For instance, we might require everyone to attend an appointment at a vaccine clinic, though they are not required to get vaccinated while they are there. This proposal stops short of mandatory vaccination in a different way from the aforementioned alternatives, but it might have certain advantages in some circumstances.

My aim here is not to advocate adoption of this mandatory attendance policy, but rather to use it in order to illustrate and clarify the range of options open to policymakers seeking to promote vaccination. I begin by reviewing the case for mandatory vaccination and then more familiar alternatives, such as incentive schemes, before turning to further explaining this mandatory attendance policy and clarifying how it differs from other proposals. If we want to know how close we can get to genuinely mandatory vaccination, then it is incumbent to consider these alternative forms of mandate, as well as alternatives to a mandate.

### Mandatory Vaccination

By mandatory vaccination, I mean that there is a requirement to be vaccinated. In particular, I am concerned with legal mandates, imposed by the state, though mandates can also be issued by other bodies, such as educational institutions or workplaces. These would still qualify as mandates so long as they are binding on subjects according to relevant norms.^2^ These bodies may not be able to impose the same sanctions as the law but, as argued below, the sanction is not necessary to the requirement.

Note that the requirements in question may be selective rather than universal; for instance a mandate may apply only to those over a certain age (Williams, 2022) or in certain occupations, such as care work.^3^ A mandate is therefore compatible with granting some exemptions. For instance, it may be binding on everyone except members of a particular religious group. However, for those who are subject to the requirement, there is no legally permissible option to refuse what is required, in this case vaccination. To be sure, this does not usually mean that they will be forcibly vaccinated against their will. As with other legal requirements, this law can be disobeyed. Thus, it is not literally true that they have no choice, but only that they have no *permitted* choice.

Further, non-compliance with a mandate will usually result in a sanction. Some authors take the threat of penalties as a defining feature of mandates (e.g. Savulescu, 2021: 81), but—while sanctions are normally needed for compliance—we could in principle have a legal requirement without any enforcement.^4^ Thus, *contra*-Giubilini and Savulescu (2019: 238), conduct is not *made* illegal by the imposition of fines or other costs. The requirement comes first and is what justifies sanctions. It is the requirement, rather than the costs attached to a breach, that characterises genuine mandates.

To be sure, this is not to say that sanctions are irrelevant. A mandate backed by a small fine (e.g. £5) is easier to justify that the same mandate backed by a much larger fine (e.g. £5000). I assume that the punishment needs to be justified, but this is independent of justifying the mandate itself. It might even be that a mandate with a small fine is easier to justify, all things considered, than a larger but non-punitive disincentive.^5^ Suppose that a special tax was imposed on those who were unvaccinated. Even if this were merely a disincentive, rather than a sanction, there may come a point at which the cost of non-vaccination was so great that people had little real choice. However, there are different kinds of freedom involved here.

Both a sanction and a price may be constraints on freedom, but these are distinct from legal obligations, which are opposed to freedom in a different sense (Miller, 1983: 79). We might see a cost as ‘conditioning’ freedom, leaving us non-free, whereas an obligation reduces freedom, making us unfree (Pettit, 2002: 347). In any case, if we are dealing with different things, then it may not be straightforward to arrive at any overall verdict as to which is best or worst all things considered. To be clear though, I am neither arguing nor assuming that mandates are always worse than other forms of interference, such as disincentives. They simply affect freedom in different ways. In the case of a disincentive, we may be *obliged* to act in a particular way, leaving us no practical choice, whereas a mandate *obligates* us, leaving us no normatively permitted choice (Miller, 1983: 79). My concern here is with the justification for mandates. I do not address what sanctions are appropriate, though this is an important further question.

There are various arguments for mandatory vaccination. Some appeal to individual benefits for those vaccinated (e.g. Giubilini and Savulescu, 2019). These arguments might be considered paternalistic though, when it comes to (younger) children, the requirement and restriction are usually on the parent. Whether or not these restrictions are ultimately justified, they are not paternalistic, because they are for the good of another (the child). It might be objected that the child is also restricted, but it is widely accepted that young children can sometimes be restricted for their own good, as well as that of others. However, debates over childhood vaccination are often entangled with those over the limits of parental decision-making (Diekema, 2011; Opel *et al.*, 2013: 1041; Pierik, 2020). I wish to avoid these added complexities here so, for simplicity, assume we are dealing with adult vaccinations.^6^ This is, of course, particularly relevant to Covid-19 vaccines, though my remarks are not specific to this case and apply to any adult vaccination.

The strongest arguments for mandates involve risks to others. Flanigan (2014) argues that we may prohibit vaccine refusal for the same reason that we prohibit celebratory gunfire: because it poses undue risk to innocent bystanders. Brennan (2018) argues that, because of over-determination, it is unlikely that any individual vaccine refuser causes harm that would not have occurred anyway. Nonetheless, he argues that we are justified in requiring vaccination because there is an enforceable duty not to participate in collectively harmful activities.^7^ Both of these arguments assume that vaccine refusers pose an active threat to others, rather than merely refusing to help. However, this distinction is not as straightforward as might be supposed. Judgements about harm depend on identifying relevant counterfactuals and may be influenced by what we think permissible (Bradley, 2012). Thus, if someone thinks that it is permissible for people to go about their business as normal, then they may conclude that this is not really an instance of causing harm and that self-isolation or vaccination (to prevent the spread) should be conceived as a non-obligatory act of benefiting others. To be clear, I am not saying that this is the correct way to draw the distinction, but only pointing out that the distinction between causing harm and refusing to benefit is itself contested.

Giubilini (2020) argues that coercive mandates are justified because fairness requires everyone to do their bit to preserve the good of herd immunity, even if any given individual’s vaccination or refusal makes no significant difference to anyone else (and therefore causes no harm). He supports this by appealing to an analogy with taxation (Giubilini, 2020: 450), though libertarians might object both to many taxes and to the idea that fairness justifies imposing duties on people without their consent (Nozick, 1974: 90–95).^8^ I do not mean to suggest that these libertarian objections are decisive, but only to highlight that Giubilini’s argument will also be contentious in some quarters, particularly amongst those most inclined to oppose government interference.

Whatever we think of these arguments, the chief obstacles to mandatory vaccination may be political, rather than philosophical. Mandatory vaccination is bound to be unpopular with significant sections of society—including some not particularly averse to vaccination itself, but hostile to government interference with their freedom.^9^ Perhaps this resistance should not be taken too seriously. People may soon become accustomed to mandates, much as most quickly came to accept seat belt laws (Giubilini and Savulescu, 2019: 244). Nonetheless, democratic governments are understandably reluctant to impose unpopular mandates, whatever their theoretical justification. The appeal of less restrictive alternatives can be explained, not only by a principled commitment to individual freedom and minimal restrictions, but also electoral self-interest. Thus, there has been considerable interest in other ways of increasing vaccination rates, without making it mandatory.

### Non-Mandatory Methods

There are a range of less coercive options for promoting vaccination. Probably the least intrusive is education, by which I mean simply imparting facts as neutrally as possible. This can increase awareness of and about vaccines, including dispelling certain fears and misconceptions. Education does not aim to change people’s minds, but simply to allow them to make informed decisions. In contrast, persuasion goes further, encouraging people to get vaccinated, for instance by portraying it as a civic duty. In practice, the line between mere education and persuasion may be hard to draw. Further, even merely educational messaging could be taken to such an extreme that it would interfere with our daily lives, for instance if citizens were constantly bombarded by public health messages (Conly, 2014: 179).

I assume that education and persuasion are relatively uncontroversial, at least in comparison to coercive measures, but they must still be used with care. There is some evidence that these interventions can backfire, actually reducing vaccination rates (Bester, 2015: 557–8). One problem here is a potential lack of trust, either in governments and/or healthcare professionals, which is an important influence on vaccination decisions (Attwell *et al.*, 2017; Jennings *et al.*, 2021). However, while (re)building trust may be imperative, it seems a mistake to contrast this and education (or persuasion), as if these are mutually exclusive alternatives. Rather, (re)building trust should be an important part of any educative/persuasive strategy.

Another possibility is to ‘nudge’ citizens towards vaccination. Nudges influence people’s choices by altering their circumstances (or ‘choice architecture’), without either prohibiting options or significantly changing economic incentives (Thaler and Sunstein, 2008: 6). This definition is so broad that educational or persuasive messaging could qualify as nudges (Sunstein, 2015: 521), though some definitions exclude rational persuasion and focus on psychological mechanisms (Kiener, 2021: 4203). In any case, nudges typically work by taking advantage of non-rational biases in people’s decision-making. For instance, if people are more likely to choose the first option offered on a menu, or stick with a default even when change is costless, then they can be encouraged to choose X rather than Y, simply by making X the first option or default. Since these methods still allow people to choose Y rather than X if they want to, advocates of nudges argue that these interventions should be acceptable even to libertarians (Sunstein and Thaler, 2003). Though much attention has focussed on paternalistic nudges, people can also be nudged to act in socially beneficial ways (Sunstein and Thaler, 2003: 1162). Given the variety of forms that nudges can take, it is difficult to generalise about the ethics of nudging. Some nudges—such as reminder notices—are unobjectionable, while others—such as deception—are problematic.

Public health messages might be framed in ways that encourage vaccination. One study found that parents are less likely to resist vaccination when physicians use presumptive, rather than participatory, language (Opel *et al.*, 2013: 1040). However, some might object that this comes too close to deception, since it might lead people to think they have no choice. It is not my aim to settle which nudges are permissible. I take it that some nudges are acceptable, though it may be that less contentious nudges have little effect, while the more effective examples raise ethical concerns of their own (Hausman and Welch, 2010; Goodwin, 2012; Wilkinson, 2013).

Incentives are another way of influencing people’s behaviour. Since such incentives are also an alternative to coercion, they are sometimes presented as a form of nudge (e.g. Blumenthal-Barby and Burroughs, 2012; White, 2018: 122; de Walque, 2020). However, nudges are supposed not to alter economic payoffs. Hence, neither taxes nor subsidies are nudges (Sunstein, 2015: 511).

Incentives, financial, or otherwise, to encourage vaccination have been widely advocated during the Covid-19 pandemic. The economist Litan (2020a, b) suggested a figure of US $1000 per individual. Anthony Albanese, leader of the Australian Labor Party, has proposed AUS $300 (roughly US $220) per person (Albanese *et al.*, 2021). A report by members of the Grattan Institute recommended a national lottery, with ten AU $1 million prizes each week (Duckett *et al.*, 2021: 38). The USA state of Ohio has actually introduced a similar lottery (Persad and Emanuel, 2021).

Savulescu (2021: 82) proposes that some form of payment could be regarded as compensation for risk, similar to remuneration for dangerous occupations such as construction work. However, whereas others have mostly focussed on cash or material goods, Savulescu (2021: 84) suggests that immunity passports might be considered ‘in-kind’ benefits. Those who get the vaccine are rewarded with greater freedom than the unvaccinated and the prospect of increased freedom, relative to lockdown, may induce people to seek vaccination. Similar ‘green pass’ proposals have been developed in Israel (Wilf-Miron*et al.*, 2021). These proposals have been characterised as ‘freedom incentives’ (McKee, 2021). They may look very much like some so-called mandates, for instance where the unvaccinated are unable to access certain public spaces such as schools or transport. However, these exclusions only amount to a genuine mandate if they are a sanction for non-compliance with a legal requirement. If there is no requirement to be vaccinated, but simply a choice between getting vaccinated and having access to the services in question or not getting vaccinated and not having access, then this is not truly a mandate but simply a (dis)incentive.

Incentives work by altering payoffs, making one option more or less attractive (Luyten *et al.*, 2011: 284).^10^Constable *et al.* (2014: 1796) propose that there should be a tax on vaccine refusal, similar to taxes on cigarettes. They suggest that this could be more effective than rewarding vaccinators, given the psychological tendency towards loss aversion.^11^ For similar reasons, they also recommend framing this cost as a penalty. However, while taxes are sometimes presented as penalties (e.g. Clarke *et al.*, 2017: 161), this risks confusing mere prices with genuine penalties. Penalties are normally sanctions in response to some rule violation, hence they express something like disapproval or condemnation. This distinguishes a fine for wrongful parking from a charge to park is a designated spot (Feinberg, 1965: 399). Similarly, a tax on tobacco differs in meaning from a fine for possession of a prohibited substance, such as cannabis. It is important to attend to the expressive difference between price incentives and penalties. Otherwise, there is a danger of sending mixed or confusing messages (Pierce, 2011: 56).

We may encourage people to choose X rather than Y either by making X more attractive (an incentive) or by making Y less attractive (a disincentive). The difference between these options is not as significant as may be supposed.^12^ Indeed, it is sometimes difficult to distinguish between them, since this depends on the assumed baseline against which they are judged. For instance, Savulescu (2021: 84) proposes vaccine passports as an incentive for vaccination, because they grant additional freedoms compared to the baseline of a universal lockdown. However, if we take the pre-pandemic normal as our baseline, this proposal could instead be viewed as an imposition for further restrictions on the unvaccinated, and hence as a cost or disincentive. While there may be a difference of framing here, it is of little (if any) normative significance. Either vaccinators are made better off or non-vaccinators are made worse off, but in both cases people are still free to choose between the options on offer, although their payoffs are altered. What distinguishes a disincentive from a sanction is that the option burdened by the additional cost remains legally permitted.

However, while this distinction between disincentives and sanctions may be clear enough in theory, it is not always so clear in practice. People sometimes react to fines—intended as sanctions—as if they were mere disincentives, and so may be *more* likely to engage in the behaviour in question (Gneezy and Rustichini, 2000). Conversely, when costs are imposed by an authoritative agent, such as the state, they may be interpreted as sanctions, even if this was not the intent (Feldman and Teichman, 2008). There is, therefore, a possibility that the messages may be misunderstood by their audience, leading to unexpected responses.^13^ Thus, while there may be an important expressive difference between these cases, public officials may have to take particular care in order to send the right messages (Lefkowitz, 2007: 222–223).

No doubt there is room for many, if not all, of these methods as part of a complete vaccination strategy. However, while these approaches may have advantages over simple mandates, they have potential problems of their own. Thus, having examined one kind of alternative to vaccine mandates, which focus on weaker (less coercive) interventions, I want to highlight a different kind of alternative. We could use a mandate that requires something short of, but related to, vaccination. If we are interested in how best to promote vaccination, or approximations to mandatory vaccination proper, then this other alternative is worth consideration.

### An Alternative Mandate

Rather than using less coercive interventions, such as nudges or incentives, we might instead employ a mandate that requires something short of actual vaccination. For instance, we might merely require people to attend an appointment at a vaccine clinic. Once there, they could be offered a jab, which they would have the right to refuse.

This ‘mandatory attendance’ policy is inspired by (so-called) compulsory voting laws. In Australia, citizens are under a legal obligation to vote in elections and can be fined if they do not. Defenders of this policy often claim that the terms mandatory or compulsory *voting* are somewhat misleading terms (Hill, 2002: 82; Elliott, 2017: 657). As a description of Australian law, this is not entirely accurate. The law *does* actually require Australian citizens to vote, not merely to attend the polls (Pringle, 2012). The secret ballot means that only attendance can be enforced in practice, but what is enforced or enforceable does not exhaust citizens’ legal obligations. Nonetheless, what many advocates of so-called ‘compulsory voting’ defend is not actually compulsory *voting*, as practiced in Australia, but merely compulsory *turnout* or *attendance* at the polling stations (Engelen, 2009; Elliott, 2017; Chapman, 2019: 102).^14^

What I have in mind is similar. Just as people may only be required to attend the polling stations, but not to vote, we could similarly require citizens to attend vaccine clinics, without actually requiring them to receive the vaccine once they are there. This is still coercive; if people do not comply with the requirement to attend the clinic, then they will face sanctions. However, what they are required to do stops short of receiving the vaccine. While this might loosely be termed ‘mandatory vaccination’, in the same way that mandatory turnout is commonly referred to as ‘mandatory voting’, what is actually mandated stops short of vaccination. Therefore, though there is still a mandate to do something, more possibilities remain open. Other things being equal, including penalties, this proposal is less restrictive of freedom than a genuine vaccine mandate.^15^ Consequently, it is likely to be more acceptable to those concerned with bodily integrity.

At first sight, this requirement may seem pointless. Mandating people to attend a clinic, but not to be vaccinated, does not obviously achieve anything. Therefore, it might be wondered what justification there could be for this. However, though its effectiveness is an empirical matter, and may vary from context to context, it is likely that this would somewhat increase the rate of vaccination. First, this will be a way of reaching those who are not necessarily opposed to vaccines, but who would otherwise put off doing anything about it. If they are forced to attend an appointment, then there is no point procrastinating any further—they may as well receive the vaccine, while they are already there. This ensures that no one misses out simply due to apathy, indifference, or being too busy. Second, there may be some who are not committed anti-vaxxers, but who are hesitant about the vaccine, perhaps due to exposure to misinformation. Their appointment will bring them into contact with medical professionals who may be able to correct false beliefs and allay irrational fears. Some of these people, once reassured, may choose to be vaccinated. While there will still be some who refuse the vaccination, even once at the clinic, it is likely that this proposal would increase vaccination compared to a wholly voluntary scheme.

Of course, there are costs to this. Forcing people to attend clinics is still a restriction of their freedom, which requires justification. Since the imposition is much less than a true vaccine mandate, it may be easier to justify. Whether or not it is justified will depend on contextual factors, such as how much this system does increase the vaccination rate and how dangerous the disease is. There may be cases where even this amount of coercion is excessive and others where this is not enough and a genuine vaccination mandate would be better. However, between these extremes, there may also be cases where a mandate only to attend a clinic strikes a reasonable balance between the state’s public health reasons to promote vaccination and individual freedom.

It might be objected that I am underestimating the costs of forcing people to attend clinics. First, the cost of attending a mandatory appointment may not be trivial for everyone. Some may find it difficult to access clinics, for instance due to remoteness, disability, lack of private transport, childcare obligations etc. These people may find an obligation to attend an appointment a non-trivial imposition. However, we already have good reasons to make vaccinations readily accessible to everyone anyway. If we are failing to do this, then a system of mandatory appointments may actually help to highlight these failings and to illustrate what can be done to address them, since those threatened with sanctions for non-attendance may appeal because, for instance, they could not easily travel to their nearest vaccine clinic. Thus, this system could produce valuable information about the barriers preventing people from getting vaccinated.

Second, it may be objected that requiring people to travel to and congregate at vaccine clinics may increase risk of virus transmission. However, any vaccination programme will involve unvaccinated people travelling to clinics. The additional risk here is only that there will be some ‘pointless’ journeys, where people attend clinics but do not get vaccinated. How much additional danger of transmission these journeys creates depends largely on how many such journeys there are, which in turn depends on how many people refuse the vaccine. However, those who refuse the vaccine do not have a strong complaint about being exposed to danger, since they have the option of vaccination if they are concerned about the risks, while those working at clinics already know that they will be coming into contact with unvaccinated people and should be provided with vaccines and personal protective equipment to minimise the risk that they face. While there may be some additional risks for others, such as public transport users, these need not be excessive.

One may still question why people should be allowed to refuse vaccination. Though people generally have particularly strong rights over their bodies, these rights are not absolute. As Flanigan (2014: 23) puts it, ‘[c]itizens do not have the right to turn themselves into biological weapons that expose innocent bystanders to undue risks of harm’. Thus, what rights we have, even over our own bodies, may sometimes be limited by other people’s rights. However, this depends on the level of danger involved. The mere fact that a choice exposes others to risks of harm does not mean that it should be prohibited. Many everyday choices that are widely considered acceptable, such as driving a car, expose others to risks (Luyten *et al.*, 2011: 283). Further, while risks to others do give us reason to intervene, these have to be balanced against the strong reasons to respect bodily integrity. Hence, we may decide that, despite the dangers that unvaccinated people may pose to others, there should be a right not to vaccinated against one’s will.

The proposal in question respects this right to refuse vaccination, since—though they are required to attend vaccine clinics—people are not required to receive the vaccine. It might be objected that this simply a variation on mandatory vaccination, rather than a genuine alternative to it. The requirement to attend an appointment at a clinic could be seen as a mild penalty for non-compliance with a vaccine mandate. However, while we *could* indeed have a mandatory vaccination programme backed by such sanctions, that is not the proposal under consideration. Recall, a mandatory vaccination programme means that there is a legal requirement to get vaccinated. But, in the mandatory attendance proposal currently under consideration, there is no such requirement. The only requirement is to attend the clinic, so those who do this have complied with their legal requirements, even if they refuse the vaccine.

While the requirement to attend an appointment at a clinic may be inconvenient, and thus a cost on non-vaccinators, this is not a penalty. A genuine mandatory vaccination scheme would impose a cost on refusers because they are in breach of a legal requirement. But, regardless of any sanction imposed, the legal requirement to vaccinate would already mean that there was no legal right to refuse. Again, this may be justified in some cases. However, if we want to recognise a right to refuse vaccination, then the scheme proposed here may be not only a genuine alternative to mandatory vaccination but also a superior one. While it does impose some cost on those who exercise their right not to be vaccinated, rights need not be costless to exercise.^16^ Crucially, the law at least permits people the option of refusal.

It might be objected that the right not to be vaccinated includes, or entails, a right not to attend a vaccine clinic.^17^ To be sure, such complaints are sometimes heard in the case of (so-called) compulsory voting laws. That is, people object to being made to attend the polling station, even if they are not actually forced to vote once they are there. No doubt, some of those opposed to vaccination would also oppose a requirement to attend a clinic. Yet, even if they still find this lesser requirement objectionable, it is surely not objectionable for the same reasons or to the same extent as a requirement to be vaccinated. Being vaccinated involves having something injected into your body which, without consent, is an invasion of one’s body. Simply being required to attend a vaccine clinic, though it affects one’s body, is not an invasion in the same way.

Of course, the requirement to attend a clinic has implications for your body, but not much more than the requirement to stay at home, which many states imposed during the pandemic. Some do indeed object to the state imposing *any* restrictions on freedom of movement. A response to those who consider any state-imposed limits on freedom intolerably objectionable is beyond the scope of this paper. I assume that the state is entitled to impose certain restrictions in the interests of public health. While there will be controversy over exactly what restrictions are justifiable, my claim is a comparative one: requiring people to attend vaccine clinics is less objectionable than requiring them to get vaccinated, since the former does not invade their body.

It might also be pointed out that many states with mandatory vaccination programmes permit at least some exemptions. There is some controversy over what grounds should justify an exemption, with some states only recognising religiously motivated refusals and others open to a wider range of conscientious objections (Lantos *et al.*, 2012; Opel and Diekema, 2012; Constable *et al.*, 2014). Nonetheless, it might be suggested that what I have proposed amounts to a mandate with exemptions available to all, albeit accompanied by the inconvenience of attending an appointment (Navin and Largent, 2017). It is true that that practical effect of these two policies may be much the same. In both cases, everyone has to attend a clinic, where they will either be vaccinated or refuse/exercise an on-demand exemption. However, these policies still differ in their symbolic or expressive meanings. If someone is exempted from a law, this means that its requirements do not apply to her. This means that she is not in breach of the requirement, but nor has she complied with it—it is simply not a requirement for her (Abizadeh, 2021). Thus, a vaccine mandate that allows some exemptions differs from my proposal for a mandate to attend a clinic, which applies to everyone without exception.

There is still a question as to which of these policies is preferable. This seems to be a matter of priorities. If we want to promote vaccination strongly, then a mandate to vaccinate with exemptions may be preferable, since this indicates that vaccination is the expected norm. However, for much the same reason, a mandate that only requires people to attend the clinic may better respect the assumed right not to be vaccinated. Under this proposed policy, people can still comply with the law—rather than requiring an exemption—while not being vaccinated. While this may be less effective at promoting vaccination, it might be better in other ways, for instance because it upholds the idea that the same laws apply equally to all. Granting some groups exemptions from certain laws has been criticised for undermining equality before the law (Barry, 1998). This provides some reason to prefer more permissive laws that apply equally to everyone, rather than stricter laws that apply to most but not all citizens.

This proposal is distinct from a genuine vaccine mandate. It could be seen as a disincentive scheme, because it imposes a burden (required attendance at a clinic) on those who refuse the vaccine. Thus, it is similar in effect to proposals imposing inconvenience on refusers (Navin and Largent, 2017). However, there are two differences. First, Navin and Largent propose attaching inconvenience to exemptions, rather than refusals. As we have just seen, the present proposal allows people to refuse the vaccine, without the need for exemptions. Second, since the requirement applies to everyone, and vaccine refusers comply with what is legally required of them so long as they attend their appointment, this may reduce the risk of the attendance requirement being misinterpreted as an inconvenience penalty (Giubilini *et al.*, 2017: 238).

Whether this proposal is all things considered preferable to more familiar alternatives, such as a true vaccine mandate or an incentive system, will depend on its consequences, which are difficult to predict and likely to vary from case to case. It is not, therefore, my aim to argue that merely mandating attendance at vaccine clinics is preferable to other options. In some cases it may be so and in others it may not. However, if we are aiming to find the right balance between promoting public health and respecting individual liberty, it is useful to consider all of the various policy options available. There may be some occasions where this policy is an appropriate balance.

### A Typology of Interventions

Though I have presented mandatory attendance as an alternative to both mandatory vaccination proper and non-mandatory methods of promoting vaccination, such as persuasion and incentives, it should be noted that these two kinds of alternatives are compatible. For instance, rather than incentivising people to get vaccinated, we could in theory incentivise them merely to attend an appointment at a clinic. Thus, there are many possible interventions, a non-exhaustive sample of which are illustrated in Table 1.

**Table 1.** A (partial) typology of vaccine-promoting interventions

	…to get a vaccine	…to attend a vaccine clinic
A legal mandate…	1. There is a legal requirement to be vaccinated. Refusers are usually liable to sanctions.	4. There is a legal requirement to attend a clinic, but not to get vaccinated. Non-attendees are usually liable to sanctions.
An incentive…	2. Those who are vaccinated receive some benefit, financial or otherwise.	5. People receive some benefit, financial or otherwise, for attending a vaccine clinic (whether or not they are vaccinated).
Persuasion…	3. People are asked or encouraged to get vaccinated.	6. People are asked or encouraged to attend a vaccine clinic.

Cell 1 represents a true vaccine mandate. One set of alternatives to this consists of interventions promoting vaccination but falling short of mandates, e.g. 2 (incentives for vaccination) and 3 (persuading people to get vaccinated). This is not a complete list; there are other possible interventions, such as nudges, which would occupy further rows.

My purpose here has been to illustrate another kind of alternative. Rather than employing less coercive means, we could instead employ coercive mandates to do something stopping short of vaccination. I have suggested a requirement to attend an appointment at a clinic, as represented in the right-hand column, though this is only one example. Again, a complete table would also feature other columns with other courses of action that people may be mandated, incentivised or persuaded to do. For instance, another possible action would be to speak to a health professional via telephone or video conference. This would likely be even further to the right of this table, since it is even less demanding than attending an in-person appointment. While this would be less of a burden, and may still deliver some of the same benefits (e.g. a chance to correct misinformation), I assume it would probably be less beneficial than an in-person appointment.^18^

Choosing a policy to promote vaccination involves choosing both a means of influence, from the left-hand side of the table (mandate, incentive, persuasion etc), *and* choosing what action people should be so influenced to do from across the top. Thus, alternatives to mandatory vaccination need not involve moving down the table, from mandates towards less coercive means of promoting vaccination.^19^ Another kind of alternative involve moving to the right, imposing genuine mandates (coercive requirements) but to do something less than vaccination.^20^ So, even if we reject mandatory vaccination proper, we could still use mandates to promote vaccination, much as mandates may be used to promote voting, even though people need not be required to cast valid votes once at the polls.

I have focussed on one particular possibility, numbered 4 on the table, i.e. a mandate to attend a vaccine clinic. However, while I have offered some arguments in favour of this combination, it is not really my aim to advocate adoption of this particular policy. What interventions are appropriate depends on the context. My main aims have been, first, to show that this is a genuinely distinct alternative to mandatory vaccination and, second, to show that it is worth taking seriously. If we think that mandatory vaccination proper would be unjustifiable, but nonetheless want something approximating it, then we should consider alternative mandates, as well as the familiar alternatives to a mandate, such as incentives or nudges.

## Footnotes

1 At least, wrongly according to the relevant normative framework. For instance, if vaccination is legally mandated, then refusal is *legally* wrong. This need not imply that it is *morally* wrong.

2 I thank an anonymous reviewer for prompting me to clarify that mandates need not be *legal* requirements, though it is legal cases that are my main concern here.

3 Mandates can also take a conditional form, for instance ‘you must be vaccinated in order to use public transport’. This could be seen as a selective mandate only for users of public transport (if you use public transport, then it is required that you be vaccinated). However, I think it is better understood as a requirement, binding on everyone, to either be vaccinated or avoid public transport. This is what Abizadeh (2021) refers to as a *wide-scope* requirement, whereas a selective mandate would be a narrow-scope requirement applying only to the relevant group. In any case, one may avoid breaking this law by not using public transport. Such a law permits people a choice, between (i) vaccination and access to public transport and (ii) non-vaccination and exclusion from public transport. Thus, it is not a *vaccine* mandate. One could alternatively have a vaccine mandate where the sanction was exclusion from public transport but, in that case, people would be in breach of the mandate whether or not they used public transport. However, in the case considered here, those who do not use public transport are complying with the law.

4
Hart (2012 [1961]: 20) points out that a command need not be backed by sanctions. What distinguishes law (or similar binding commands) from a mere threat, such as that issued by an armed robber, is the claim to authority. The robber employs force, rather than right, whereas the state’s directives at least purport to be binding.

5 I thank an anonymous referee for presenting me with this objection. Consider three governments, which introduce the following policies: A introduces a mandate, backed by a £5 penalty. B introduces a mandate, backed by a £5000 penalty. C introduces a £5000 tax on the unvaccinated (no mandate). I assume B’s policy is hardest to justify. A’s is easier to justify because, although A and B each impose a mandate, A imposes smaller penalties. On the other hand, C imposes the same cost, but does so without a mandate. The mandate and the cost each require justification, but they are two different things. Comparison between A and C is not straightforward, even though both are clearly easier to justify than B.

6 Although the same considerations may also apply to older children, once they are able to give and refuse consent and to be held legally responsible for their actions. I take no particular stance on the point at which they become appropriate.

7 Brennan’s ‘clean hands principle’ is criticised by Blunden (2019) and Giubilini (2020: 449).

8 To be clear, libertarians need not object to all taxes. For instance, Nozick argues that a minimal state is justified in protecting people’s rights *and* in charging for this protection. However, he would object to how most (if not all) actual states raise and spend tax revenue. I thank an anonymous reviewer for prompting me to clarify this.

9 Though Lewandowsky (2021) suggests that libertarian political beliefs are associated with science denial. However, not all libertarians are anti-vaxxers, as demonstrated by Brennan (2018). Indeed, Butler and Sorell (2022) argue that many appeals to libertarianism, in order to justify vaccine refusal, are disingenuous. For further discussion of libertarian responses to the present Covid-19 pandemic, see Cato and Inoue (2022).

10 An anonymous referee points out that we may want to distinguish between benefits or costs that are deliberately imposed in order to encourage vaccination and those that are incidental side effects of other policies. Those of the latter sort may have a similar effect, but they are ‘unintended incentives’.

11 Thus, while the imposition of the cost itself is not a nudge, framing it as a loss for the unvaccinated, rather than a gain for the vaccinated, is a nudge.

12 This is particularly the case when it is the state acting, since an incentive payment would likely be funded by taxes anyway, thereby imposing a cost on non-vaccinators.

13 Note that nothing here commits me to identifying what was expressed with what the agent *intended* their words or actions to express. We may express things that we do not intend. My point is only that the audience’s (mis)interpretation of an expression does not alter what was expressed.

14 Though there are parallels between mandatory vaccination and mandatory voting, one might favour compulsion in one case but not the other. For instance, Brennan (2018) argues for mandatory vaccination but Brennan (2014) rejects mandatory voting.

15 An anonymous reviewer points out that mandatory vaccination backed by a small penalty may be less restrictive overall than mandatory attendance backed by a large penalty. However, this comparison involves two different things. A large penalty is more restrictive than a small one, while a more permissive mandate (attendance rather than vaccination) is less restrictive.

16 For an analogous case, see Lefkowitz (2007, 2018). He argues that the state should respect a right to civil disobedience, but that this is compatible with imposing costs (which he characterises as penalties rather than punishment) on the exercise of that right. I prefer to avoid the language of penalties, but on the substantive issue—whether imposing costs on doing X is consistent with respecting the right to do X—Lefkowitz and I are in agreement.

17 I thank an anonymous referee for this objection, though Lever (2008: 64, n. 4) presents a parallel argument against compulsory voting, employing the analogy that a requirement to attend church still violates one’s freedom of conscience, even if one is not actually required to pray.

18 First, because talking to someone via telephone or video conference is less likely to generate trust than talking to them face-to-face and, second, because there is no opportunity for someone to get the vaccine there and then.

19 Note that this table distinguishes different *kinds* of intervention (e.g. a mandate with sanctions, incentives etc). It does not consider the size of the sanction, incentive etc in question. We may think not only that a mandate with large sanctions is more coercive than a mandate with small sanctions, but also that a mandate with small sanctions is less coercive overall than a large (dis)incentive. If this is so, then options on lower rows are not always less coercive than those on higher rows. I thank an anonymous referee for this observation.

20 We might also move right *and* down. For instance, we might incentivise people to attend a vaccine clinic (possibility 5 on the table). This option may be less attractive, but it is another possibility.
